# Impact of Grazing on Diversity of Semi-Arid Rangelands in Crete Island in the Context of Climatic Change

**DOI:** 10.3390/plants11070982

**Published:** 2022-04-04

**Authors:** Maria Karatassiou, Zoi M. Parissi, Sampson Panajiotidis, Afroditi Stergiou

**Affiliations:** 1 Laboratory of Rangeland Ecology, School of Forestry and Natural Environment, Aristotle University of Thessaloniki, P.O. Box 286, 54124 Thessaloniki, Greece; afroster@for.auth.gr; 2 Laboratory of Range Science, School of Forestry and Natural Environment, Aristotle University of Thessaloniki, P.O. Box 236, 54124 Thessaloniki, Greece; pz@for.auth.gr; 3 Laboratory of Forest Botany—Geobotany, School of Forestry and Natural Environment, Aristotle University of Thessaloniki, P.O. Box 270, 54124 Thessaloniki, Greece; pansamp@for.auth.gr

**Keywords:** aridity index, effective number of species, Shannon entropy, richness, Gini–Simpson

## Abstract

The rangelands of Crete island (Greece) are typical Mediterranean habitats under high risk of degradation due to long-term grazing and harsh climatic conditions. We explored the effect of abiotic (climatic conditions, altitude) and biotic factors (long-term grazing by small ruminants) on the floristic composition and diversity of selected lowland (Pyrathi, Faistos) and highland (Vroulidia, Nida) rangelands. In each rangeland, the ground cover was measured, and the floristic composition was calculated in terms of five functional groups: grasses, legumes, forbs, phrygana, and shrubs. The aridity index, species turnover, species richness, Shannon entropy, and Gini–Simpson index (with the latter two converted to the effective number of species) were calculated. Our results reveal that highlands are characterized by the highest aridity index (wetter conditions). Lowland rangelands, compared to highland, exhibited a higher percentage contribution of grasses, legumes, and forbs, while species turnover decreased along the altitudinal gradient. The Shannon entropy index was correlated (a) positively with Gini–Simpson and mean annual temperature and (b) negatively with mean annual precipitation, aridity index, and altitude. Moreover, the Gini–Simpson index correlated positively with mean annual temperature and negatively with altitude. Our results could help to understand the effects of grazing on rangeland dynamics and sustainability in semi-arid regions in the context of climatic change.

## 1. Introduction

Arid and semi-arid rangelands occupy approximately 40% of the Earth’s land surface and influence the livelihood and well-being of one-fifth of the world’s human population [1,2]. More than one billion people rely on rangelands for their living, primarily through extensive livestock production, and roughly two billion acquire animal protein, water, or other resources from these biomes [3,4]. Rangelands comprise many habitats and host economically important species offering support to approximately 50% of the world’s livestock, providing forage production for both domestic and wildlife populations [5,6,7].

Despite their high importance, most of the non-marketed services of these rangelands and their economic value have often been neglected [8,9]. Moreover, they have faced increased risks resulting from overutilization and degradation [10,11,12]. The estimated extent of rangeland degradation varies extensively, from as little as 10–20% to as much as 70–80% [3]. Desertification is a cumulative threat that includes both climatic and land-use drivers that interact in space and time [13].

It is well demonstrated that rangelands are maintained by grazing. However, they can be severely affected by the high intensity of the latter, climate change, soil quality, nutrient depletion, fire, habitat fragmentation, as well as human activities [4,7,14]. In most rangelands, precipitation [15] and grazing [16,17] are the most important factors determining species diversity and ecosystem function [18,19]. On the other hand, altitude, which greatly affects the abiotic environment by modifying climatic variables and the topography [20,21], is an indirect gradient that is correlated with resources and regulators of plant growth [22,23] and species composition [24].

Although it is known that the richness of vascular plant species decreases with an increase in altitude [25,26,27], the patterns of response are rather fickle [28]. Changes in plant species richness along altitudinal transects are of great importance in the study of global climate change [29,30]. The spatial change in species composition involves the study of beta diversity and species turnover. Because of the greater diversity of habitat conditions, mountains have higher levels of species turnover than lowland areas [31], and under a climate change scenario, mountains are considered significant for the maintenance of biodiversity [32,33,34]. The relationship between climatic conditions and species turnover is described by the relationship between climatic factors and regional species richness [35].

To assess the impact of grazing on vegetation, the effect of precipitation on species diversity should be thoroughly studied and considered [36]. Both low ground cover and plant diversity increase the vulnerability of rangelands to climate change [37,38]. Overgrazing, which is prescribed as a decrease in productivity [39] and loss of biodiversity [40,41], is considered one of the main causes of land degradation in arid and semi-arid regions worldwide [42]. Heavy grazing directly changes the floristic composition of plant communities selectively, changing the structure and composition of communities at the expense of palatable species [43,44], and may also indirectly modify the outcome of competitive interaction by changing light availability [45]. The impact of grazing intensity on plant diversity varies along the precipitation gradient [46,47].

There is a list of methods employed to study diversity, which is a multi-dimensional phenomenon [48]. The simplest measure of diversity is to calculate the number of species (richness) in an area, which, however, does not take into consideration species abundances and is sensitive to sample size. Other approaches consider species abundance (Shannon index) or give weight to dominant species (e.g., Gini–Simpson). The Shannon and Gini–Simpson measures of diversity are themselves mere indices and not “true” diversities [49,50,51,52,53]. The true diversity of an investigated community is simply the community of equally common species (effective number of species, ENS) required to give the same value of an index calculated for the community in question [52,54,55]. In recent years, the use of ENS has been established in ecological studies. After the conversion of classical indices (Shannon and Simpson) to ENS, diversity is always measured in the number of species, providing more interpretable and comparable assessments of diversity [54,56,57].

Thirty-five percent of the Greek land, and more specifically 37–50% of the land in Crete, is characterized as critically susceptible to desertification due to the combination of a warming climate with low precipitation and intensified human activities [58,59]. To the best of our knowledge, the effect of grazing and climatic conditions on grassland biodiversity has not yet been studied in Crete, a vulnerable Mediterranean region.

The current study aimed to investigate the effect of abiotic (climatic conditions, altitude) and biotic factors (long-term grazing) on the floristic composition and diversity of lowland and highland rangelands on the island of Crete, Greece, which are typical Mediterranean habitats at high risk of degradation. We aimed to answer the following questions:

(a) Do patterns of species diversity indices and composition differ among rangelands exposed to different grazing intensities?
(b) Do these differences vary among rangelands with different altitudes and climatic conditions?

## 2. Results and Discussion

The current study indicates that the existing high grazing pressure, in combination with climatic conditions, could result in rangeland degradation on Crete island.

**Figure 1:**
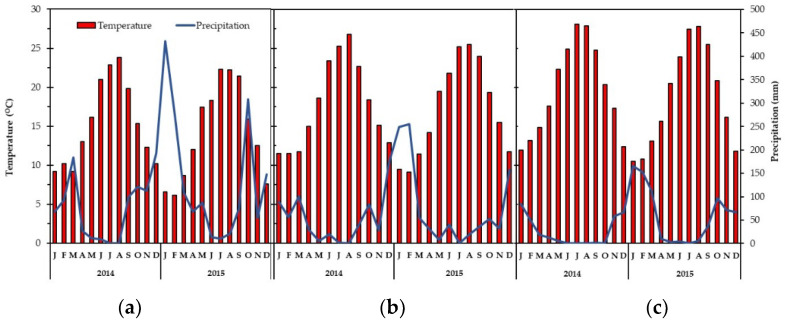
Monthly means of air temperature (°C) and precipitation (mm) at (**a**) Vroulidia—Nida, (**b**) Pyrathi, and (**c**) Faistos rangelands during the experimental period.

Diverse climatic conditions prevail among the four studied rangelands (Figure 1). At Faistos and Pyrathi, the mean annual temperature was 19.17 ± 1.24 and 17.38 ± 1.18 °C, and the mean monthly precipitation was 45.77 ± 11.05 and 60.67 ± 15.1 mm, respectively. At Vroulidia and Nida, an inverse trend was observed as the mean annual temperature was 12.67 ± 1.98 °C with an average monthly precipitation of 107.05 ± 23.93 mm. Pyrathi had higher rates of precipitation and mean monthly temperature compared to Faistos (Figure 1b,c). The climatic data indicated a shorter drought period in Vroulidia and Nida, which implied that plant species faced a water deficit for a shorter period in these areas. 

**Figure 2:**
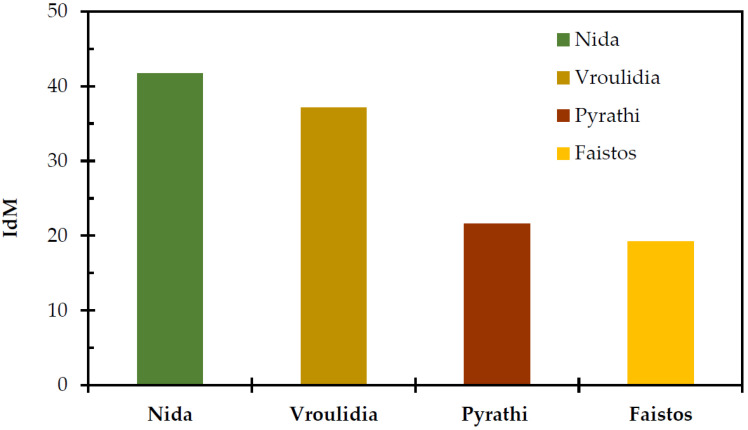
Martonne’s aridity index (I_dM_) for the studied rangelands for the period 1979–2013.

The aridity index (I_dM_) classifies the type of climate in relation to water availability, and it is a crucial environmental factor affecting the growth of natural vegetation. In the present study, I_dM_ was negatively correlated with mean air temperature and positively with altitude and precipitation (Table 1). The Nida rangeland scored the highest aridity index, followed by Vroulidia, while Faistos had the lowest one (Figure 2). The higher values of I_dM_ in the highlands indicated higher humidity [60] and better climatic conditions for plant growth and development. Mallen-Cooper and coauthors [61] found similar results in eastern Australia, which support that aridity decreases when the height of precipitation and absorptivity of water increase. The higher I_dM_ correlated with the higher available water resources over time and, consequentlym lower vulnerability to desertification.

The aridity level interacts with plant traits related to stress resistance to determine the floristic composition and vegetation responses to domestic animal grazing [62,63,64,65,66]. The impact of grazing on species richness and composition under high-aridity conditions could be either high [67] or low [65].

Generalized Linear Model analysis showed significant differences (*p* < 0.001) in forage production in both fenced plots and grazed sites and for FUP (Table 2) among the studied rangelands. Additionally, there was a significant interaction between rangeland and year (*p* < 0.001), but only for forage production in grazed sites. The forage production in fenced plots ranged from 189.2 ± 24.2 to 116.5 ± 7.3 g m^−2^, while in grazed from 128.9 ± 4.8 to 15.8 ± 1.4 g m^−2^. The lowland rangelands had higher forage production in relation to the highland. This is in agreement with Bhandari and Zhang [68], who demonstrated that altitude is negatively related to aboveground biomass. Concerning the year, it was a significant predictor (*p* < 0.001) only for FUP. The FUP was 84.4–87.1% at Nida, 74.1–76.1% at Vroulidia, 74–75% at Pyrathi, and 15–17% at Faistos for 2014 and 2015, respectively (Table 2). The highest value of FUP was presented in Nida and the lowest in Faistos. In many cases, the high FUP is related to low vegetation percentage cover, a result of overgrazing [69].

It is known that the cover of vegetation is a health indicator of the rangelands. Our data analysis revealed significant differences (*p* < 0.05) in vegetation cover among the studied rangelands. The vegetation cover recorded in Nida and Vroulidia scored the lowest values, 52% to 66% and 64% to 70%, for 2014 and 2015, respectively, in comparison to the lowland sites of Pyrathi (78–90%) and Faistos (92–99%). The low vegetation cover and the high FUP at Nida and Vroulidia are likely the results of overgrazing, as the number of transhumant small ruminants, and, consequently, the grazing pressure is immoderate in the Psiloritis mountain [70]. Papanastasis and coauthors [71] point out that the Psiloritis mountain is overgrazed as the stocking rate is four times higher than the grazing capacity. Ojima and coauthors [72] found that overgrazing results in the loss of vegetation cover and increased erosion. The low vegetation cover provides low protection from soil erosion and a high risk for degradation [13] and reduced soil porosity [46,73]. This reduced vegetation cover and the lower plant diversity probably increase the susceptibility of rangelands to the effects of climate change as well [37,38,46].

**Figure 3:**
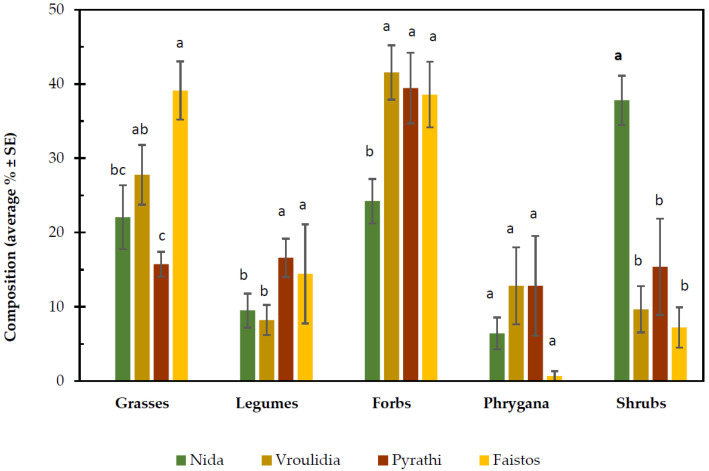
Floristic composition per functional groups (grasses, legumes, forbs, phrygana, shrubs) (%) at the four studied rangelands for the experimental period. Values represent means ± SE (n = 6). Different letters in columns indicate significant differences for the same parameter (*p* < 0.001).

Data analysis revealed that in all functional groups, there were no significant differences between years and no significant interaction between rangeland and year (*p* ≥ 0.05). On the contrary, there was a significant interaction between rangeland and functional groups (*p* < 0.001) (Table 3, Figure 3). Overall, lowlands, compared to highlands, present a significantly (*p* < 0.05) higher percentage contribution of grasses, legumes, and forbs (Figure 3). On the other hand, shrubs had a significantly (*p* < 0.05) higher percentage in the highlands compared to the lowlands, while phrygana [74,75] had similar participation in both lowlands and highlands. Concerning the contribution of functional groups separately in highland and lowland, shrubs were significantly (*p* < 0.05) higher at Nida compared to Vroulidia, while the opposite trend was detected for forbs in highlands for both experimental years (Figure 3). As others point out as well, the Cretan landscape, especially in high elevations, is a mixture of woodland and open vegetation, where many woody species are found in various forms (from trees to small or dwarf shrubs) [76,77,78]. Many of these shrubby taxa are shaped by grazing and are adapted to this pressure, which includes prescribed fires. Moreover, woody plants are able to ‘colonize’ rocky places where soil can be scarce. Moreover, Papanastasis and coauthors [77] found that woody species on the Philoritis mountain cover 30% of the soil. Regarding the lowlands, there is significantly higher participation of grasses (*p* < 0.05) in the Faistos rangeland compared to Pyrathi (Figure 3). On the contrary, the participation of phrygana was higher at Pyrathi compared to Faistos.

On Crete island, as elsewhere in Greece, farmers traditionally improve grassland productivity [79] and quality through fire management of vegetation, mainly phrygana, which enables the modification of the floristic composition. The fires decrease the percentage of shrubs and phrygana and drive the ecosystem to a previous successional stage (secondary succession), where the percentage of grasses and legumes is higher, leading to higher herbage biomass production in terms of quantity and quality [80,81]. The floristic composition of the studied rangelands is strongly linked to habitat characteristics (abiotic factors: altitude, climatic conditions) and primary consumers (biotic factors) [82].

**Figure 4:**
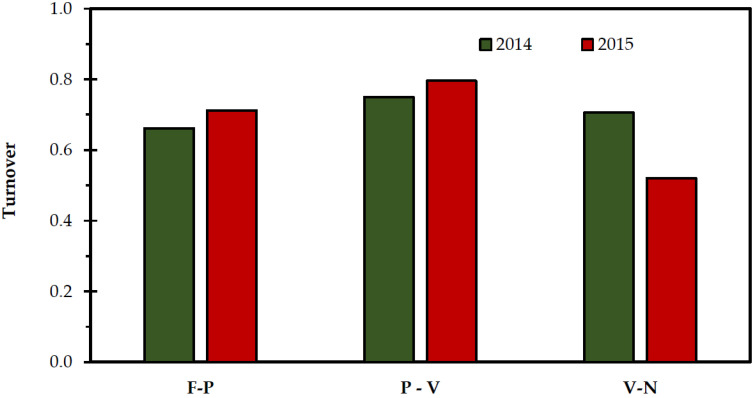
Species turnover in the studied rangelands Faistos—Pyrathi (F-P), Pyrathi—Vroulidia (P-V) and Vroulidia—Nida (V-N) for the experimental period.

A drop in species turnover is observed between pairs of lowland to highland rangelands (Figure 4). Species turnover presented the highest value at an intermediate altitude from 355 to 1100 m a.s.l.; and decreased at higher altitudes, from 1100 to 1530 m a.s.l., due to the range of ecological adaptation and growth of plants at different altitudes. The same results were found by Mena and Vázquez-Domínguez [83] when the species turnover in mammals was studied, more specifically, small rodents, along an altitudinal gradient. Our results support the hypothesis that species turnover decreases with altitude only at the higher altitudinal zone. The lower species turnover in highlands could be attributed to the presence of sparse vegetation in mountainous areas generally [79], and probably, species turnover correlates with different rangeland management [84].

**Figure 5:**
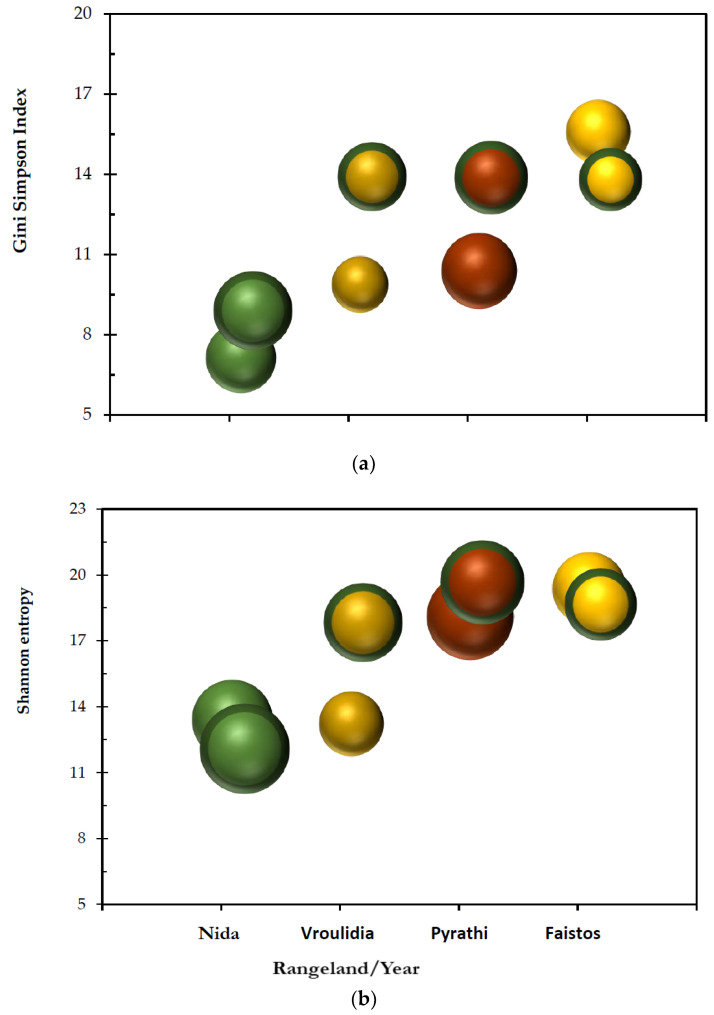
(**a**) Gini–Simpson vs. richness index and (**b**) Shannon entropy vs. richness index for all studied rangelands for the experimental period, 2014 and 2015 (symbols encircled). Cycle size is proportional to richness. Gini–Simpson and Shannon entropy are given as effective numbers of species (ENS).

Diversity in terms of abundance (ENS Shannon entropy) was lower than species richness, while diversity in terms of dominance (ENS Gini–Simpson index) was lower than the Shannon entropy for both years of the study (Figure 5). This result indicated that there is species dominance in all study areas. The greater the dominance in the community, the greater the differences among these three parameters [51,52]. In both years of the study, the species richness, Shannon entropy, and Gini Simpson were higher in the lowlands of Faistos and Pyrathi compared to Vroulidia, while at Nida, the highest species richness was recorded. It is noteworthy that there are more plant species at Pyrathi than at Faistos, and the same trend for diversity (Figure 5). This was contrary to the theory that species richness and diversity decreased with an increase in altitude. The species richness may not be related to altitude, as it is demonstrated by Zawierucha and coauthors [85]. This unexpected result is probably due to the fire set by shepherds to improve forage production at Pyrathi last year and led to a change in the floristic composition. It has been proven that fire has important effects on diversity and plant community composition [86,87,88,89,90]. Shannon entropy could be used in situations where rare and abundant species or traits are expected to be equally important [91]. However, if dominant species or traits are expected to be more essential, then Gini–Simpson would be more relevant. Both indices were smaller than richness for all rangelands (Figure 5), as they were based more on abundant and dominant species, respectively [82]. Although the environmental conditions favored plant growth in mountainous areas, the grazing pressure significantly decreased species diversity. Nida had the highest species richness in relation to the other studied rangelands, but its species abundance (Shannon entropy) and dominance presented the lowest value. These results could be attributed to high FUP (overgrazing), lower vegetation cover, soil erosion, and unpalatable plant species encroachment that will be exacerbated by climate change [92].

Heavy grazing (high FUP) may result in high-level species replacement [93]. Plants at Pyrathi and Vroulidia are grown under different climatic conditions but under similar FUP, presented different species richness but similar diversity in terms of species abundance and dominance. At Faistos, under light grazing pressure, the rangeland presented similar high ratios of abundant and dominant species to total species recorded (richness). These results could be verified from the ratios of Shannon entropy/richness and Gini Simpson index/richness (Table 4). The highest ratio was recorded at Faistos and the lowest one at Nida. Faistos, with the longer semi-arid period under low FUP, presented a very diverse vegetation pattern, with three-quarters of all species showing the same abundance, while more than half were also dominant (Table 3). As grazing intensity escalates from Pyrathi to Nida, ratios of abundance diversity/richness and dominance diversity/richness decrease; this is more evident in terms of absolute ENS values of abundance and dominance diversity (Figure 5). Vroulidia shows higher ratios than Pyrathi but similar absolute ENS values for dominance diversity and lower for dominance diversity. These rangelands, without these disturbances (climate, grazing), would gradually decline due to the successional process to the next successional stages [94,95]. Animal grazing is a key factor in avoiding the successional processes of vegetation [82].

According to the Pearson correlation coefficient, the Shannon entropy index was positively correlated with the Gini–Simpson and mean annual temperature and negatively with mean annual precipitation and altitude, while the Gini–Simpson index correlated negatively with altitude (Table 1). According to the results, the species diversity decreased with an increase in altitude and precipitation. This is in agreement and supports the theory that species richness and diversity decrease along the altitude gradient [27,96,97]. Altitude probably has the strongest effects on species richness, abundance, and ground cover [98]. Nevertheless, other studies found that overgrazing affects functional diversity more than climate, and species diversity declines with an increase in grazing intensity in areas with different climatic conditions [41,46,99]. It is well known that the relationships between diversity indices do not always follow mathematically predicted patterns [100,101].

## 3. Materials and Methods

### 3.1. Study Area

**Figure 6:**
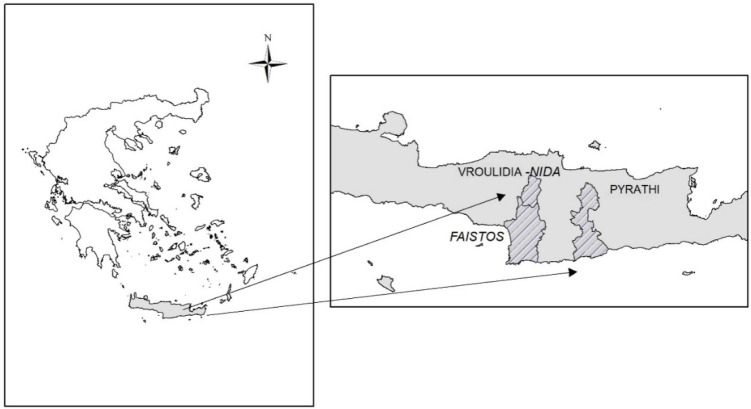
The experimental rangelands on the island of Crete, Greece.

The research was conducted during 2014–2015 on the island of Crete, the southernmost part of Greece. The selected experimental sites were two lowland rangelands of Heraklion prefecture: Faistos (F) (24°51′20″ E, 35°06′30″ N) 155 m a.s.l. and Pyrathi (P) (25°11′21″ Ε, 35°05′52″ Ν) 355 m a.s.l., and two in the highlands of Psiloritis mountain (Rethymnon prefecture): Vroulidia (V) (24°47′02″ Ε, 35°10′58″ Ν) 1100 m a.s.l. and Nida (N) (24°50′33″ Ε, 35°12′48″ Ν) 1530 m a.s.l. that have been subjected to grazing (Figure 6).

The livestock farming system was introduced on the island about 8000 years ago, and animal husbandry has been used by humans to transform natural ecosystems to produce more grazing material and, therefore, more animal products for their own consumption and survival. Through these processes, the extensive forests of the island were turned into rangelands, while the abandoned fields due to grazing could not be reforested. Uncontrolled and random, both spatially and temporally, grazing is the rule on the island. In lowlands, e.g., Faistos, in recent years, a change in land-use has been observed with farming replacing pastoralism, so there is low grazing intensity in the area. On the other hand, the lowlands of Pyrathi are heavily grazed all year round by sheep and goat flocks. Concerningly, the highlands of Vroulidia are grazed all year by sheep and goats, while Nida, from April to October, by transhumant small ruminant flocks. The highlands are characterized by a long history of small ruminant overgrazing [71].

The climate of the lowland and highland rangelands is characterized as Csa and Csb, respectively, in the Köppen–Geiger system (www.en.climate-data.org, 12 December 2021). The daily climatic data (precipitation, average temperature) for the two lowland rangelands (P, F) (Figure 1b,c) were obtained from the nearest meteorological stations, while for the highlands (N, V) (Figure 1a) from the only one available meteorological station located between them, and are reported as mean monthly data for the period in which the study was conducted.

### 3.2. Field Data

The vegetation (ground) cover was measured at the end of the growing season according to the line and point method [102]. Three experimental transects (25 m each) [103,104] were established in each rangeland, as the habitats were homogeneous. After that, the floristic composition was calculated and presented in five functional plant groups: (1) grasses, (2) legumes, (3) forbs, (4) phrygana, and (5) shrubs, according to their life form and by distinguishing legumes from forbs based on their nutritional value for small ruminants (Table S1). Moreover, two sampling quadrats of 0.35 x 0.35 m were established in every transection of each rangeland at 8 and 16 m in order to calculate: (a) species richness (equivalent to its own numbers) and (b) species diversity indices (Shannon entropy and Gini–Simpson), which were converted to the effective number of species (ENS). Shannon entropy was calculated following the formula in Equation (1) below

(1) $$H=-\sum_{i=1}^{S}p_{i}lnp_{i}$$

and was converted to ENS by taking its exponential exp(H) (exponential of Shannon entropy index), where p_i_ is the population frequency of the ith species. The Gini–Simpson index (H_GS_) was converted by the transformation

1/(1−H_GS_),

which is the inverse of the index

$$1/(\sum_{i=1}^{s}pi^{2})$$

[51,52,57,105]. These measures easily pass interpretable counts and provide information at three different levels based on how rare and abundant taxa are weighted [53,105,106,107].

For every studied rangeland, the aridity index (de Martonne index, I_dM_) was calculated following the formula in Equation (2) below [60]:

I_dM_ = P/(T + 10) (2)

where P is the mean annual precipitation (mm), and T (°C) is the mean annual air temperature. The values of T and P for every rangeland were downloaded from Climatologies, at high resolution (30 × 30 s), for the Earth’s land surface areas (CHELSA, http://chelsa-climate.org/, 2 February 2022), which is a global climate database covering the period 1979 to 2013.

The species turnover was calculated as the gain and loss of species between altitudes following the formula in Equation (3) below [108]:

β(H) = (g(H) + l(H))/(α(H) + α(H-1))(3)

where g(H) and l(H) are the number of species gained and lost, respectively, from altitude H-1 to altitude H, while α(H) and α(H-1) is the species richness at altitude H and H-1, respectively [108].

In order to estimate the forage utilization percentage (FUP) in the spring of 2012 in each of the four rangeland’s three plots, 9 m^2^ were fenced to be protected from grazing. The above-ground herbage production was collected by clipping three 0.5 × 0.5 m quadrats in each fenced plot (i.e., nine quadrats per fenced plot). In the same period into grazed rangelands (sites), the remaining above-ground biomass after grazing was collected by clipping in three similar quadrats in each transect (i.e., nine quadrats per rangeland), in May 2014–2015. Consequently, grazing intensity was expressed by FUP. The difference among herbage yields of fenced (UG) and grazed sites (G) was used to calculate FUP from the formula of Equation (4) below [109]:

FUP= [(UG–G)/UG] × 100(4)

### 3.3. Statistical Analysis

The Generalized Linear Model (GLM), assuming a normal distribution, was used to assess whether the altitude of each rangeland, functional group, and year were significant predictors of ground cover and floristic composition. Before analysis, the data were converted to ln + 1 to meet assumptions of normality (tested with the Kolmogorov–Smirnov test) and homogeneity of variances (Levene’s test). Estimated marginal means for all the above factors were calculated with pairwise contrasts, and LSD adjustment was applied for the multiple comparisons (α = 0.05). The data in the figures and tables depict values before the transformation. Pearson correlation was used to explore links among R, SE, GS, I_dM_, T, P, and altitude. All statistical analyses were performed using the SPSS statistical package v. 27.0 (IBM Corp. in Armonk, NY). The Paleontological statistics software package for education and data analysis (Past) was used to calculate the diversity indices.

## 4. Conclusions

The Mediterranean basin includes a wide range of vegetation, climatic, and edaphic conditions that have been shaped by natural selection under the pressure of a distinct climate and human activities. Our results demonstrate the strong relationship between diversity and temperature and agree with the fact that vegetation diversification is strongly related to the climatic gradient and is more related to temperature than precipitation. Moreover, this research could help to understand how grazing intensity and climatic conditions interactively influence rangelands dynamics in semi-arid regions and monitor the livestock management and decision making in these areas.

## Figures and Tables

**Table 1:** Pearson correlation between richness (R), effective Shannon entropy (SE) and Gini–Simpson (GS), Martonne aridity index (I_dM_), mean annual temperature (T), mean precipitation (P), and altitude for the four studied rangelands.

	R	SE	GS	I_dM_	T	P	Altitude
R	1						
SE	−0.073	1					
GS	−0.280	0.861 **	1				
I_dM_	0.059	−0.876 **	−0.678	1			
T	−0.295	0.894 **	0.795 *	−0.954 **	1		
P	−0.174	−0.766 *	−0.498	0.947 **	−0.809 *	1	
Altitude	0.218	−0.888 **	−0.763 *	0.980 **	−0.986 **	0.882 **	1

* Significant for *p* < 0.05, ** Significant for *p* < 0.001.

**Table 2:** Forage production (g m^−2^) in fenced plots and grazed sites and forage utilization percentage (FUP %) in the four studied rangelands in the study years. Values represent means ± SE (n = 9). Different letters in the same column indicated significant differences (*p* < 0.05).

	Forage Production (g m^−2^)				FUP (%)	
	Fenced Plots		Grazed Sites			
Rangeland (R)	2014	2015	2014	2015	2014	2015
Nida	122.2 ± 6.03 b	120.2 ± 5.5 bc	15.8 ± 1.4 d	18.8 ± 1.1 d	87.1 ± 0.9 a	84.4 ± 0.5 a
Vroulidia	126.7 ± 9.5 b	116.5 ± 7.3 c	29.9 ± 1.7 c	30.2 ± 2.2 c	76.1 ± 0.8 b	74.1 ± 1.0 b
Pyrathi	189.2 ± 24.2 a	173.6 ± 22.2 a	45.7 ± 6.5 b	42.2 ± 4.9 b	76 ± 0.7 b	75.1± 1.8 b
Faistos	134.4 ± 4.0 b	151.4 ± 5.3 b	111.5 ± 3.3 a	128.9 ± 4.8 a	17.00 ± 0.8 c	14.9 ± 0.8 c
R	<0.001 **		<0.001 **		<0.001 **	
Year	0.748 ns		0.084 ns		0.003 *	
R X Year	0.567 ns		0.017 *		0.761 ns	

* Significant for *p* < 0.05, ** Significant for *p* < 0.001, ns not significant.

**Table 3:** General linear model analysis for the effects of rangeland and year on participation of functional plants groups.

	Wald Chi-Square	df	*p*-Value
Year	0.033	1	*p* ≥ 0.05 ns
Rangeland	0.045	3	*p* ≥ 0.05 ns
Functional group	116.816	4	<0.001 *
Year * Rangeland	0.014	3	*p* ≥ 0.05 ns
Rangeland * Functional group	80.120	12	<0.001 *

* Significant for *p* < 0.001, ns not significant.

**Table 4:** Ratios of Shannon entropy/richness (HE/R) and Gini–Simpson (GS/R) index/richness for all of the studied rangelands for the experimental period.

	Lowland Rangelands				Highland Rangelands			
	Faistos		Pyrathi		Vroulidia		Nida	
	2014	2015	2014	2015	2014	2015	2014	2015
* HE/R	0.74	0.75	0.50	0.58	0.66	0.59	0.43	0.31
* GS/R	0.60	0.55	0.29	0.41	0.49	0.46	0.23	0.23

* Gini–Simpson and Shannon entropy are given as effective number of species (ENS).

## Data Availability

The data presented in this study are available in the figures and tables provided in the manuscript.

## Supplementary Materials

The following are available online at https://www.mdpi.com/article/10.3390/plants11070982/s1, Table S1: Plant species in floristic composition at the four studied rangelands (life form: Grass (G), Legume (L), Forb (F), Phrygana (PH), Shrub (S).
