# Supplementary material for: Impact of Grazing on Diversity of Semi-Arid Rangelands in Crete Island in the Context of Climatic Change
Source: Plants (Basel). 2022 Apr 4;11(7):982. doi: 10.3390/plants11070982 (PMC9003301; doi:10.3390/plants11070982)
Supplement: Supplementary file 1 [file plants-11-00982-s001.zip › plants-1553620-supplementary.pdf]

**Table S1**. Plant species in floristic composition at the four studied rangelands (life form: Grass (G), Legume (L), Forb (F), Phrygana (PH), Shrub (S).

| Life form | Species | Rangelands | | | |
|---|---|---|---|---|---|
| | | Nida | Vroulidia | Pyrathi | Faistos |
| S | *Acer sempervirens* L. | X | X | | |
| F | *Achillea cretica* L. | X | | | |
| G | *Aegilops caudata* L. | | | X | X |
| G | *Aegilops geniculata* Roth | | | | X |
| G | *Aegilops ovata* L. | | | | X |
| G | *Aegilops umbellulata* Zhuk. | | | X | X |
| G | *Agrostis stolonifera* L. | | | | X |
| F | *Anagallis arvensis* L. | | X | X | |
| S | *Anchusa cespitosa* Lam. | | | X | X |
| F | *Anthemis chia* L. | X | X | | |
| F | *Anthemis cretica* L. | | | | X |
| G | *Anthoxanthum odoratum* L. | X | | | |
| F | *Arum italicum* subsp. *concinnatum* (Schott) K. Richt. | | X | | |
| L | *Astragalus creticus* Lam. | | | | X |
| G | *Avena fatua* L. | | | X | |
| G | *Avena sterilis* L. | | | X | X |
| F | *Bellardia viscosa* (L.) Fisch. & C.A. Mey. | | | X | X |
| F | *Bellis perenne* L. | X | | | |
| S | *Berberis cretica L.* | X | | | |
| G | *Brachypodium distachyon* (L.) P. Beauv. | | | X | X |
| G | *Bromus hordeaceus* L. | | X | X | X |
| G | *Bromus intermedius* Guss. | | | X | |
| G | *Bromus japonicus* Thunb. | | X | X | X |
| G | *Bromus madritensis* L. | | | X | |
| G | *Bromus rigidus* Roth | | X | | |
| G | *Bromus squarrosus* L. | X | X | | |
| G | *Bromus sterilis* L. | X | | X | |
| G | *Bromus tectorum* L. | | | X | |
| PH | *Calicotome villosa* (Poir.) Link | X | | X | |
| F | *Capsella bursa-pastoris* (L.) Medik. | X | X | | |
| F | *Carlina corymbosa* L. | X | X | X | |
| F | *Centaurea raphanina Sm.* subsp. *Raphanina* | X | X | | |
| F | *Centaurium erythraea* Rafn | | | X | |
| F | *Cerastium brachypetalum* Pers. | X | X | | X |
| F | *Cerastium glutinosum* Fr. | | X | | |
| F | *Cichorium pumilum* Jacq. | | | X | |
| F | *Cichorium spinosum* L. | X | | X | |
| PH | *Cistus creticus* L. | X | | X | X |
| F | *Clinopodium vulgare* subsp. *orientale* Bothmer | X | X | | |
| S | *Crataegus monogyna* Jacq. | X | X | | |
| F | *Crepis sancta* (L.) Bornm. | X | X | | |
| F | *Crepis sibthorpiana* Boiss. & Heldr. | | | X | X |
| G | *Crithopsis delileana (Schult.)* Roshev. | X | X | X | |
| F | *Crucianella angustifolia* L. | | | | X |
| F | *Cyclamen creticum* Hildebr. | X | | | |
| G | *Cynodon dactylon* (L.) Pers | X | X | | |
| G | *Cynosurus echinatus* L. | X | | | |
| G | *Dactylis glomerata* L. | X | X | X | |
| F | *Dianthus juniperinus* subsp. *bauhinorum* (Greuter) Turland | X | | X | |
| F | *Dianthus Xylorrhizus* Boiss. & Heldr. | | | | |
| F | *Draba muralis* L. | | | | |
| L | *Ebenus cretica* L. | | | X | |
| F | *Echium parviflorum* Moench | | | | X |
| G | *Elymus farctus* (Viv.) Melderis | | | X | |
| S | *Erica arborea* L. | | | X | |
| F | *Eryngium campestre* L. | X | X | X | X |
| F | *Eryngium ternatum* Poir. | X | | X | |
| S | *Erysimum mutabile* Boiss. & Heldr | X | | | |

| | | | | | |
|---|---|---|---|---|---|
| S | *Euphorbia acanthothamnos* Boiss. | | X | | |
| F | *Ferula communis* subsp. *glauca* (L.) Rouy & E.G. Camus | | | | X |
| G | *Festuca arundinacea* Schreb. | | X | | |
| G | *Festuca ovina* L. | X | X | | |
| F | *Foeniculum vulgare* Mill. | | | | X |
| F | *Galium aparine* L. | | X | | |
| F | *Galium incurvum* Sm. | X | X | | |
| F | *Galium murale* (L.) All. | | | | X |
| S | *Genista acanthoclada* DC. *genista* | X | | X | |
| F | *Geranium molle* L. | X | X | | |
| F | *Geranium purpureum* Vill. | | X | | |
| F | *Glebionis coronaria* (L.) Spach | | | X | |
| S | *Globularia alypum* L. | X | X | | |
| S | *Helichrysum stoechas* (L.) Moench | X | | | |
| F | *Helminthotheca echioides* (L.) Holub | X | X | X | X |
| F | *Hieracium schmidtii* Tausch | | | X | |
| F | *Holosteum umbellatum* L. | | X | | X |
| G | *Hordeum bulbusum* L. | X | | X | X |
| G | *Hordeum marinum* Huds. | | | | X |
| G | *Hordeum murinum* L. | | X | X | |
| L | *Hymenocarpos circinnatus* (L.) Savi | X | | X | |
| F | *Hypericum perforatum* L. | | | X | |
| F | *Hypochaeris tenuiflora* (Boiss.) Boiss. | | | X | |
| L | *Lathyrus sphaericus* Retz. | | | X | |
| F | *Leontodon tuberosus* L. | | | X | |
| G | *Lolium perenne* L. | | | X | X |
| L | *Lotus corniculatus* subsp. *tenuis* (Willd.) Berher. | | | X | X |
| L | *Medicago lupulina* L. | X | X | X | X |
| L | *Medicago minima* (L.) Bartal. | | | X | X |
| L | *Medicago sativa* L. | | | | X |
| G | *Melica ciliata* L. | | | | X |
| L | *Melilotus albus* Medik. | | | X | X |
| F | *Muscari comosum* (L.) Mill. | | | X | |
| F | *Myosotis ramosissima* Rochel | | X | | |
| S | *Olea europaea* L. | | | X | |
| L | *Onobrychis sphaciotica* Greuter | | | X | |
| F | *Oxalis pes-caprae* L. | | | | X |
| F | *Pallenis spinosa* (L.) Cass. | | | | X |
| G | *Phalaris aquatica* L. | | | X | |
| S | *Phillyrea latifolia* L. | | X | | |
| G | *Phleum subulatum* (Savi) Asch. & Graebn. | | | | X |
| S | *Phlomis cretica* C. Presl | | | | X |
| PH | *Phlomis lanata* Willd. | X | | | |
| F | *Plantago lanceolata* L. | X | X | X | X |
| G | *Poa bulbosa* L. | X | X | X | |
| G | *Poa trivialis* L. | X | X | X | X |
| F | *Polygonum idaeum* Hayek | | | X | X |
| S | *Prasium majus* L. | X | | | |
| F | *Prunella cretensis* Gand. | | | | X |
| F | *Ranunculus sprunerianus* Boiss. | X | X | | |
| G | *Rostraria cristata* (L.) Tzvelev | X | X | | |
| S | *Rubus sanctus* Schreb. | | X | | |
| F | *Rumex acetosella* L. | | X | | |
| F | *Rumex pulcher* L. | | X | | |
| PH | *Salvia fruticosa* Mill | | | | X |
| F | *Sanguisorba cretica* Hayek | | | | X |
| PH | *Sarcopoterium spinosum* (L.) Spach | X | X | X | |
| PH | *Satureja thymbra* L. | | | | X |
| F | *Scandix pecten-veneris* L. | | X | X | X |
| F | *Scilla nana* (Schult. & Schult. f.) Speta | | X | | |
| S | *Securigera cretica* (L.) Lassen | | | | X |
| F | *Sherardia arvensis* L. | X | | | |

| | | | | | |
|---|---|---|---|---|---|
| F | *Silene variegata* (Desf.) Steud. | | X | | X |
| F | *Silybum marianum* (L.) Gaertn. | X | | X | |
| F | *Sinapis arvensis* L. | | | | X |
| F | *Sisymbrium officinale* (L.) Scop. | | X | | |
| G | *Taeniatherum caput-medusae* (L.) Nevski | | | X | X |
| S | *Teucrium alpestre* Sm. | X | | | |
| S | *Thymelaea hirsuta* (L.) Endl. | | | X | |
| S | *Thymus leucotrichus* Halácsy | | | X | |
| L | *Trifolium alexandrinum* L. | | | X | X |
| L | *Trifolium angustifolium* L. | | | X | X |
| L | *Trifolium campestre* Schreb. | X | X | X | X |
| L | *Trifolium fragiferum* L. | | | X | X |
| L | *Trifolium glomeratum* L. | X | X | | |
| L | *Trifolium hybridum* L. | X | | | |
| L | *Trifolium nigrescens* Viv. | | X | X | X |
| L | *Trifolium physodes* M. Bieb. | X | | | |
| L | *Trifolium stellatum* L. | | X | | |
| L | *Trifolium tomentosum* L. | X | | X | |
| L | *Trifolium uniflorum* L. | X | X | | X |
| F | *Valerianella echinata* (L.) DC. | | | X | |
| L | *Viola alba* subsp. *cretica* (Boiss. & Heldr.) Marcussen | X | | | |
| F | *Viola fragrans* Sieber | X | | | |
| F | *Vulpia myuros* (L.) C.C. Gmel. | | X | X | |
